# Identification of universal grass genes and estimates of their monocot-/commelinid-/grass-specificity

**DOI:** 10.1093/bioadv/vbaf079

**Published:** 2025-04-07

**Authors:** Rowan A C Mitchell

**Affiliations:** Sustainable Soils and Crops, Rothamsted Research, Harpenden, Hertfordshire AL5 2JQ, United Kingdom

## Abstract

**Motivation:**

Where experiments identify sets of grass genes of unknown function, e.g. underlying a QTL or co-expressed in a transcriptome, it is useful to know which of these genes are common to all grasses (universal) and whether they likely have monocot-/commelinid-/grass-specific function.

**Results:**

A pipeline used data on 16 grass full genomes from Ensembl Plants to generate 13 312 highly conserved, universal groups of grass protein-coding genes. Validation steps showed that 98.8% of these groups also had gene matches in recently sequenced genomes from two major grass clades not used in the pipeline. Comparison with many non-grass genomes identified 4609 of these groups as likely of monocot-/commelinid-/grass-specific function. Both grouping of genes and specificity were defined using hidden Markov model (HMM) profiles of the groups. The HMM-based approach performed better than simple percentage identity in discriminating between test sets of known specific and non-specific genes. The results give novel insight into the nature of monocot-/commelinid-/grass-specific genes. Researchers can use the universal_grass_peps database to gain evidence for their experimentally identified grass genes being involved in monocot-/commelinid-/grass-specific traits.

**Availability and implementation:**

The universal_grass_peps database is available for download at https://data.rothamsted.ac.uk/dataset/universal_grass_peps.

## 1 Introduction

Grasses (*Poaceae*) dominate open habitats in which they played a fundamental role in forming due to characteristics of fast growth and resilience (Linder *et al.* 2018). These make them suited to adoption in agriculture and today, about 70% of the calorie intake and 30% of protein for humans comes directly or indirectly from grasses. Key grass adaptations to open habitats include: morphology that allows meristems to avoid consumption and fire damage allowing regrowth; tissues rich in silica to resist herbivory and stress (Mitani-Ueno and Ma 2021); stomata that can respond faster than those of other plants to rapidly changing conditions of open habitats (Chen *et al.* 2017); cell walls containing ferulate implicated in lowering digestibility and stress resistance (Chandrakanth *et al.* 2024); unique inflorescence and seed characteristics for efficient reproduction (Kellogg 2001). These traits are the result of specific protein-coding genes, non-coding genes, and regulatory genomic elements that arose in the evolution of grasses; the pipeline described here is designed to identify the protein-coding genes (henceforth referred to as “genes” for brevity) involved in grass-specific traits.

**Figure 1.**
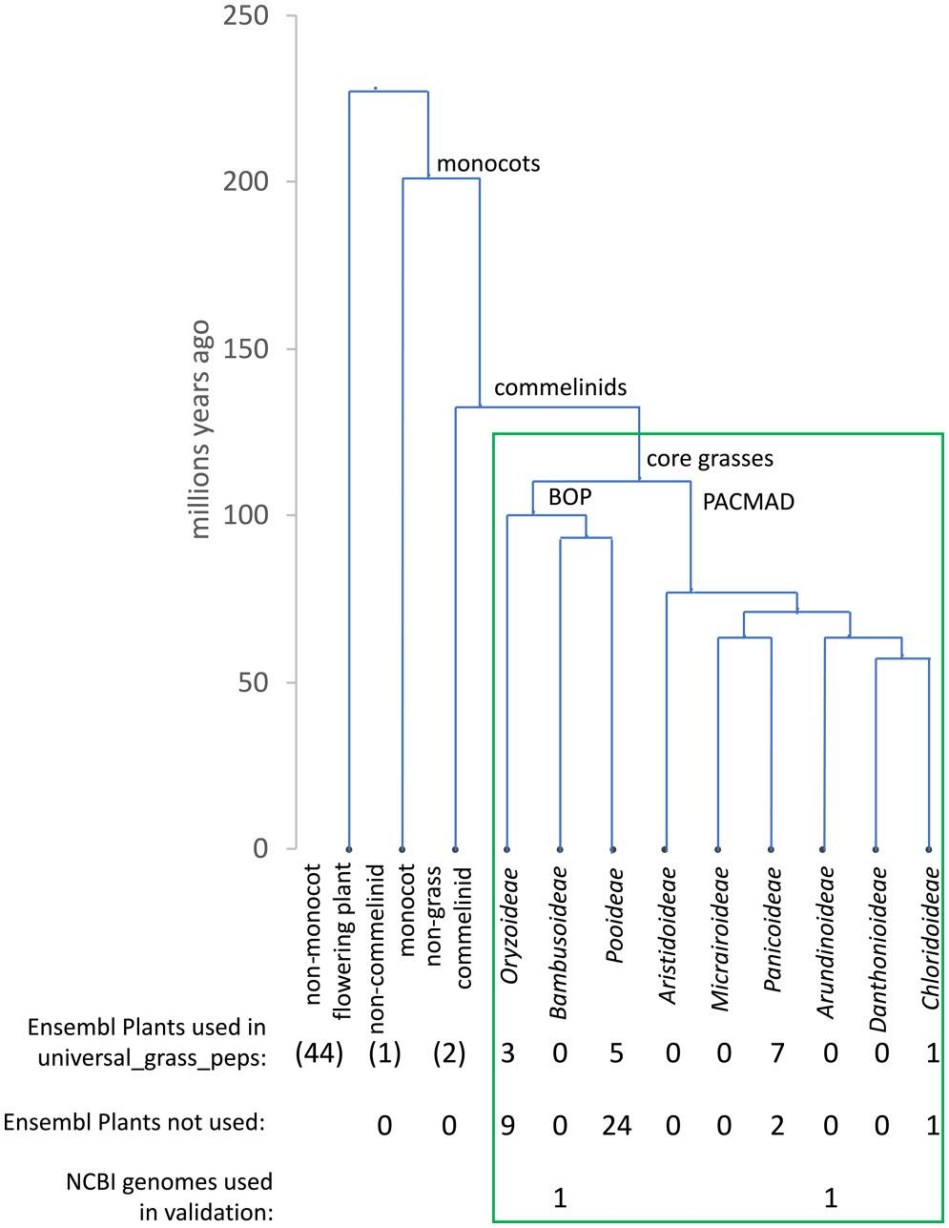
Phylogenetic tree of monocots, commelinids, and grasses with estimated divergence times from Zhang *et al.* (2024). The core grass species inside the green box are those represented in the universal_grass_peps database, with those outside used to test specificity. Numbers of fully sequenced genomes from Ensembl Plants release 56 used and not used in the pipeline and from NCBI for validation are shown for each taxon.

Relatively few genes involved in these traits have been demonstrated experimentally but some key examples are listed in Table 1. It can be postulated that these grass genes and others responsible for functions specific to monocots/grasses that are key to grass fitness will be (1) present in all grasses, i.e. universal, (2) highly conserved, (3) have no close homologs in species outside monocots. The concept of universality of genes—matching genes being present in all organisms within a taxonomic unit—is a useful guide to their importance for fitness and implicitly groups genes by function (Kriventseva *et al.* 2019). On point (3), it is convenient to consider monocot- and grass-specificity together because the large number of non-monocot plant genomes and wealth of gene knowledge (particularly for *Arabidopsis*) make for a better reference set than the few, less studied non-grass monocot genomes. Also many key gene functions may have evolved first in monocots and then been expanded by gene duplication in grasses. Thus, the aim is to capture those genes with key functional innovations that arose in monocots or grasses and have not diverged further within the core grasses. Figure 1 shows a phylogenetic tree of monocots and grasses with estimates of divergence times from Zhang *et al.* (2024). From this timescale, these functional innovations would have occurred in the period between 230 and 110 million years ago.

**Table 1.** Categories of monocot-/commelinid-/grass-specific genes and examples of these.

Category	Gene name(s)	Description	References
Responsible for commelinid/grass specific feature of cell wall	*OsCslF2; CSLF6*	Synthesis of (1,3; 1,4)-β-glucan	Burton *et al.* (2006)
	*FSFT, SFT1-4; PMT, FMT, AT10*	Addition of hydroxycinnamates to precursor for addition to xylan, or to monolignols	Yang *et al.* (2024); others reviewed in Chandrakanth *et al.* (2023)
	*XAT2, XAT3; XAX1*	Addition of arabinofuranose or hydroxycinnamoyl-arabinofuranose to xylan	Anders *et al.* (2012) Feijao *et al.* (2022)
	*EXPB9*	β-Expansins mediate expansion in grass primary cell walls	Sampedro *et al.* (2015)
Control of grass-specific reproductive morphology	*CFO1*	Regulates floral organ identity	Sang *et al.* (2012)
	*ramosa2*	Responsible for genetic control of grass-specific inflorescence	Bortiri *et al.* (2006)
	*AMD1*	Regulates tapetum development	Zou *et al.* (2022)
	*OsDAF1, OsINP1*	Regulate pollen aperture	Zhang *et al.* (2020)
	*NSG1*	Regulates spikelet development	Zhuang *et al.* (2020)
	*MOF1*	Regulates spikelet development	Ren *et al.* (2020)
	*FZP*	Regulates panicle branching	Bai *et al.* (2017)
	*OsETT2*	Promotes awn development	Yamaguchi *et al.* (2004)
Control of grass-specific vegetative morphology	*LG2, LG2L*	Determine leaf lamina joint positioning	Wang *et al.* (2021)
	*DL*	Midrib development	Yamaguchi *et al.* (2004)
Transporter for commelinid/grass specific Si uptake/distribution	*LSi1, LSi6*	Transporters required for active uptake and distribution of Si	Ma and Yamaji (2015)
Involved in commelinid/grass specific stomatal opening	*SLAC1*	Nitrate-sensitive guard cell anion channel	Schäfer *et al.* (2018)
Component of grass-specific strategy for Fe uptake	*DMAS1*	Synthesis of deoxymugineic acid	Bashir *et al.* (2006)
	*OsYSL18*	Fe(III)–deoxymugineic acid transporter	Aoyama *et al.* (2009)

To find optimal grouping of grass genes into groups of universal genes requires several innovative steps, as major bioinformatic resources (e.g. Ensembl, Phytozome) group genes by evolutionary relationships, i.e. as orthologs or paralogs, making no attempt to identify which have diverged in function. Orthologs and paralogs are identified by most likely evolutionary path of mutation over whole sequence, whereas what matters for shared function is those elements in the encoded peptide that affect the function, identifiable in peptide sequences by high conservation (Capra and Singh 2007). Hidden Markov models (HMMs) which give greater weight to conserved sequence elements important for function have long been used as a highly effective means of comparing and functionally categorizing peptide sequences (Yoon 2009). Therefore, HMM match scores should be a better criterion for grouping universal genes of common function and for comparing other genes to these groups. To account for the greater likelihood of orthologs sharing function at a given level of sequence similarity (Gabaldón and Koonin 2013), orthologs should be selected rather than other genes with same HMM score; however paralogs can occasionally displace orthologs to perform same function so this possibility should ideally be allowed for in a pipeline.

Previous studies have identified lineage-specific genes, e.g. in *Brassicaceae* (Donoghue *et al.* 2011) or primates (Shao *et al.* 2019) using cut-off scores from pairwise comparisons (by blastp or whole-genome alignments). These cut-off values are chosen conservatively so that the lineage-specific genes are highly likely to be new function, but the pipelines do not explicitly consider whether they have diverged in function within the lineage. The aim of the pipeline presented here is different as it is only concerned with universal genes of putative common function within the grasses. This allows a novel approach where a HMM profile can be built from all the genes within a universal group and used to search the non-grasses to judge specificity.

Using these principles a novel bioinformatics pipeline was designed which aims to: (a) identify a maximal set of groups of highly similar genes found in all grasses with each group having putative common function, (b) assign estimates of how specific these functions are to monocots/commelinid-/grass species based on closest hits from species outside these taxa.

## 2 Methods

### 2.1 Selection of sequenced genomes

The Ensembl Plants database (Bolser *et al.* 2016) release 56 (https://feb2023-plants.ensembl.org/), which was used as the source of sequence and ortholog information, has fully sequenced genomes of grasses in four of the main taxa of core grasses (Fig. 1). From these, 16 grass species of the most important crops (including rice, wheat, barley, *Lolium*, rye, sugar cane, sorghum, millet, maize) and models (*Brachypodium distachyon* and *Setaria viridis*) were chosen for input to the pipeline with eight each from the BOP and PACMAD clades that together comprise the core grasses. Fifty-eight non-grass species genomes were also used for specificity steps in pipeline and details of all Ensembl genomes used are given in Supplementary Table S1. Whilst the highest-level BOP and PACMAD clades are represented in Ensembl Plants, there are no genomes from some major grass clades one level down (Fig. 1) which could lead to bias in identification of universal grass genes. To address this, two recently sequenced genomes of grasses within *Bambusoideae* and *Arundinoideae* clades available on NCBI were used in validation steps.

### 2.2 Pipeline overview

**Figure 2.**
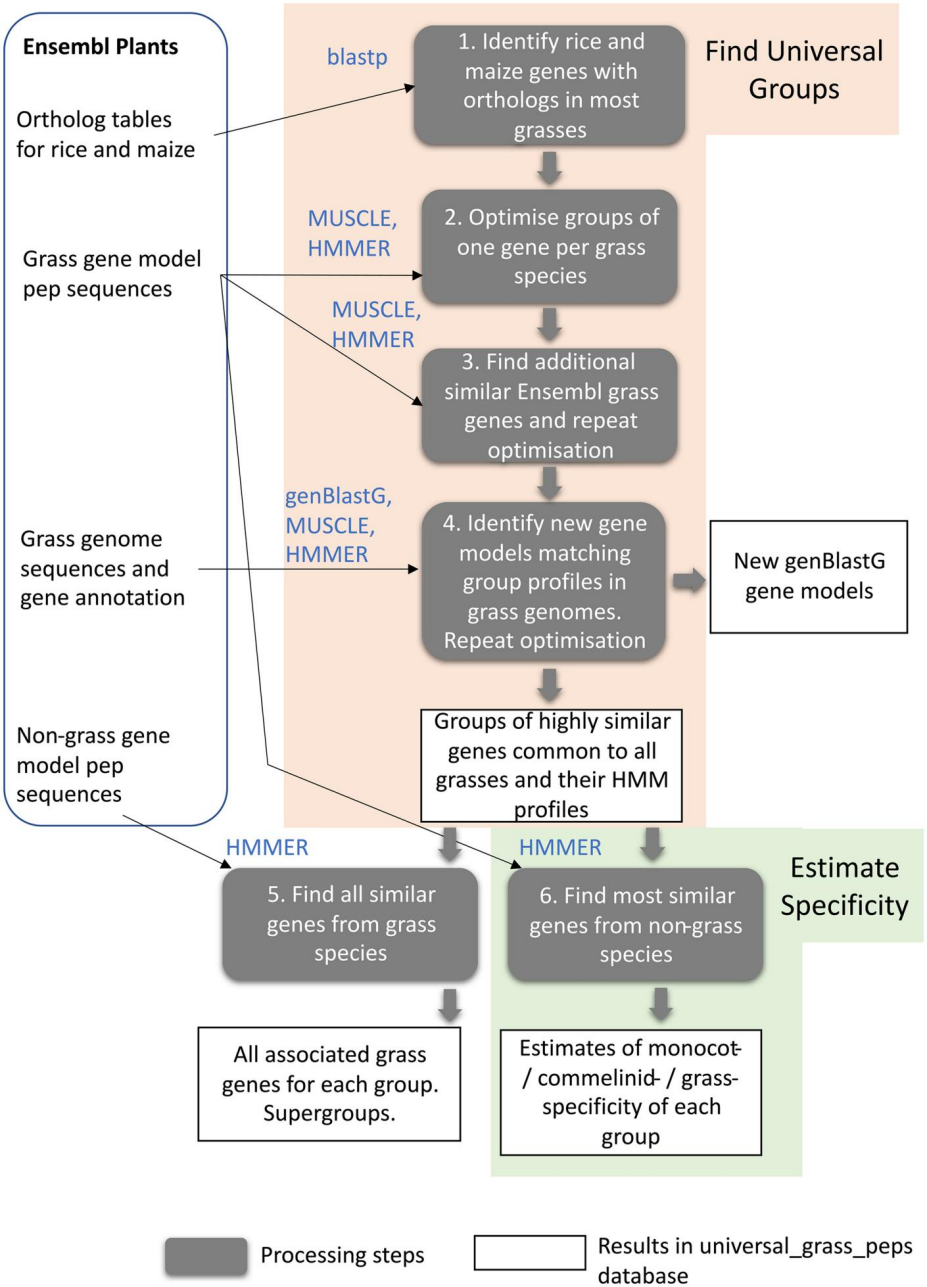
Pipeline that generates the database of highly conserved universal grass protein-coding genes and estimates of their monocot-/commelinid-/grass-specificity (universal_grass_peps). All the input data is taken from Ensembl Plants release 56 and the processing steps are carried out by custom scripts, using the external tools shown in blue text, to generate universal_grass_peps database.

Figure 2 shows a scheme of the pipeline which takes input data downloaded from Ensembl Plants, processes these using custom software and public packages and generates datasets that populate a novel database called universal_grass_peps. The following input data were manually downloaded from the Ensembl Plants database: peptide sequences (peps) from gene models for 16 grass species and 58 non-grass species, the full genome sequences of the grasses with their gene annotations, and the ortholog tables of rice and maize gene models to all other grasses (downloaded using the Biomart tool). Rice and maize were chosen as the reference species because they are intensively studied crops with well annotated genomes representing respectively the BOP and PACMAD clades.

All operations on these input data were carried out on the Rothamsted Linux High Performance Cluster (912 CPU Core, 16TiB memory) using custom Perl (with Bioperl routines; Stajich *et al.* 2002) and bash scripts to run bioinformatics tools and process data. These scripts are available at https://github.com/Rowan-ACM/universal_grass_peps. The complete pipeline took 11 days of run time on the cluster to complete. The methods used in the different components of the pipeline shown in Fig. 2 are described below.

### 2.3 Identification of highly conserved peptides present in all grasses

Membership of universal groups was determined by iteratively trying different peps to get the best HMM score (optimization) in groups with one pep per grass species as described below (Find Universal Groups block in Fig. 2). First, some preparatory steps were needed to find all potential peps to be considered in the optimization and these were taken from the ortholog tables for the rice and maize reference species. Initially, groups were formed with one reference species entry and multiple candidate peps for other grass species.

Using peps from gene models of the 16 grass species, any identical ones were removed but all non-identical peps from splice variants were retained. The next step was to populate groups with the closest orthologs to each rice and maize pep. To reduce the complexity of this step, very similar maize and rice peps were collapsed into “clusters” defined as >90% identical in both directions of a pairwise comparison for all comparisons within a cluster. [Using blastp from BLAST+ package (Camacho *et al.* 2009) parameters: -evalue 1.e-5 -max_target_seqs 50 -seg no -max_hsps 1]. An ortholog table from the Ensembl multiple tables was then defined where each entry was defined by a primary key (group ID) of the rice peptide or peps cluster ID. Where there was no maize ortholog, the most similar maize pep was found with blastp and if this was not orthologous to another rice gene, added along with all the other grass genes orthologs of the maize gene to the group and group ID was set to composite of rice and maize seed cluster IDs. Groups from maize were also allowed where there was no rice ortholog or similar pep sequence (as this could be added by the later genBlastG step). Other grass species orthologs to rice and/or maize were assigned exclusively to a single group based on highest ranking by Ensembl ortholog confidence flag (0 or 1; defined from tree-compliance and, in a small number of cases, whole-genome alignment and gene order conservation; https://plants.ensembl.org/info/genome/compara/peptide_compara.html) then sequence similarity. Groups which had no entries for more than four of the 16 grasses were deleted and gene members orthologous to remaining groups were reassigned according to this ranking. At this stage (Box 1, Fig. 2) there were groups of multiple genes per grass all of which were classed orthologous to rice and/or maize. Using two reference species in this way allows for similar non-orthologous genes of potentially common function to be grouped together due to descending from two paralogs. But by using the ortholog information orthologous peps were more likely to be assigned to same group than non-orthologs with same level of similarity in accordance with the principle that orthologs are more likely to share function at a given level of sequence similarity (Gabaldón and Koonin 2013).

The next step (Box 2 in Fig. 2) was to optimize membership of groups keeping only one peptide sequence per species. This condition is required to meet the principle that each universal group represents a unique function required for fitness; it also makes the profile scores comparable across all groups and avoids biasing profile to species with many members in a group. HMM profiles of each group were initially generated using the top ranked pep sequence for each species. To make HMM profiles, all the group member peps were aligned using MUSCLE v3.8.1551 with default parameters (Edgar 2004) then the HMM profile was generated from this multiple alignment with hmmbuild (parameters—amino—fragthresh 0) and hmmpress commands from HMMER package version 3.3.2, November 2020 (Eddy 2022). Similarity scores of the member sequences against their own profile were obtained using hmmscan (all hmmscan steps in pipeline used parameter E 1.e-7, other parameters default). To compare across groups, this score was normalized to a maximum possible score obtained with the consensus sequence of the profile (generated by hmmemit command) to derive a HMM relative score (*R*). Then group members were each substituted with all the alternative peptide sequences for this group and species; if *R* was improved by >0.01 the substitution was kept as the group member; this requirement means that peptide sequences ranked as best orthologs in previous step tended to be kept as group members. It was found that groups could be further improved by using grass Ensembl gene models hits to the HMM profile found with hmmscan that were not members of other groups; these are peps not found by previous steps probably because they were not in ortholog tables. Again, these peps were assigned as group members if they improved *R* by >0.01 (Box 3 in Fig. 2).

In the next step (Box 4 in Fig. 2), the genBlastG tool was used (She *et al.* 2011) which searches for gene models with canonical splice junctions in genomic sequence using a query peptide sequence; here the consensus from HMM profile for the group was used as the query. For each group, and for each grass where the current member was missing or low scoring, the relevant grass genome was searched with genBlastG (v138, parameters -p genblastg -v 2 -h 0 -j 3 -r 1 -norepair). Any hits discovered by genBlastG were checked that they were novel by comparing exon coordinates with those of all Ensembl gene models using gff files. Using criteria as above, if a novel gene model from genBlastG improved the profile, it was adopted as the group member for that species and the HMM profile was rebuilt. A maximum of four genBlastG gene models were adopted so every profile has at least 12 Ensembl gene models. At the completion of this process, the *R* value was recalculated for each member and groups where the lowest scoring member had *R* < 0.65 or had missing members were discarded; the cut-off of 0.65 is a criterion for high conservation and the value was selected as that for which 90% of the expected universal non-specific genes (Supplementary Table S2; see below) groups passed (see Section 4.2 for more on this parameter).

HMM profiles from the complete set of groups that pass these were compiled into a single HMMER database, the universal_grass_peps HMM database.

### 2.4 Comparison with use of OrthoFinder

OrthoFinder was assessed as an alternative source of ortholog information to input to the pipeline which has reportedly better performance in prediction of orthologs than Ensembl method (Emms and Kelly 2019). Using the non-redundant pep fasta files for the 16 grasses, orthogroups were generated by OrthoFinder (version 2.5.5, parameters -S blast, others default). Membership of universal_grass_peps groups was compared with these orthogroups.

### 2.5 Validation steps with genomes from outside Ensembl Plants

Independent support for rice genBlastG gene models generated by the pipeline was tested by comparison with gene models in the more recent IC4R rice annotation curated at https://ngdc.cncb.ac.cn/ic4r/ (Sang *et al.* 2020). Top IC4R hits for each genBlastG gene model query were identified by blastp with *E* < 1.e−5; hits covering >90% of pep on correct chromosome were taken as matches.

Of the major grass clades not represented in Ensembl Plants, two (*Bambusoideae* and *Arundinoideae*) have genomes recently available on NCBI (Fig. 1). One of these is an annotated genome for *Phragmites australis* an *Arundinoideae* species, assembly accession GCF_958298935.1 (Christenhusz and Fay 2024). There are no annotated bamboo genomes but there is a high-quality unannotated genome for bamboo species *Phyllostachys violascens*, assembly accession GCA_044048585.1. The *P. australis* gene model peps were used as queries against the universal_grass_peps HMM database and groups which had hits with *R* > 0.65 were taken as matches. Groups not already matched in *P. australis* and all groups for *P. violascens* were used to search for genBlastG hits in the genome sequences of these species as described above for main pipeline; the genBlastG gene models found were used to obtain *R* score for each group HMM.

### 2.6 Matches of grass genes to universal groups

All scores for Ensembl grass peps against the universal_grass_peps HMM database for all groups were obtained (Box 5 in Fig. 2). All non-members that had scores of *R* > 0.65 to any group were allocated as associate peptides allowing many-to-many relationships (this allows a look-up search with any peptide ID as query to find all groups to which a peptide is similar). To check whether some universal_grass_peps groups can be regarded as likely same function, the *R* of grass group members against other group HMMs were obtained. Where all members of a group scored >0.65 for another group and vice versa these groups were allocated to a supergroup of potential same function.

### 2.7 Monocot-/commelinid-/grass-specificity

To help define specificity and derive a cut-off value for the specificity metric in the pipeline, two sets of genes were defined using external evidence (Estimate Specificity block in Fig. 2). For the non-specific test set, a list of proteins of known function expected to be common across all plants was compiled. This non-specific set was derived from ribosomal subunit proteins using RPG database (Nakao *et al.* 2004) (http://ribosome.med.miyazaki-u.ac.jp) and enzymes or enzyme subunits in amino acid synthesis, glycolysis, photosynthetic electron transport, CBH cycle, and nucleotide synthesis from OryzaCyc database which had identical steps in AraCyc database within Plant Metabolic Network (Hawkins *et al.* 2021) giving a total of 240 rice peptides (Supplementary Table S2). For the specific gene set, monocot-/commelinid-/grass-specific protein-coding genes of known function (Table 1) were identified from the literature. The papers were from prior knowledge or from a literature search for the terms “monocot-specific” or “grass-specific”, or from a search for grass-specific morphology (e.g. “awn development”, “ligule”, “panicle branching”) in gene descriptors in the RAP-DB database of rice genes (Sakai *et al.* 2013). These genes were added to the specific set where the publication showed good evidence of direct involvement in control of grass-specific morphology. The resulting specific gene set outlined in Table 1 is listed in more detail in Supplementary Table S3 and contains evidence on specificity from 25 publications for 31 genes.

Scores were obtained for the best-matching non-monocot peptide sequence from all the 55 non-monocot species against universal_grass_peps HMM database for all groups. A metric of monocot-/commelinid-/grass-specificity *S* for each group was evaluated, defined as *R* of the lowest scoring grass member of this group minus *R* of highest scoring non-monocot peptide. This method considers variation in conservation between groups so a hit to a highly conserved group will require a higher HMM score to give the same *S* score as a hit to a less conserved group. By definition a value of *S* ≤ 0 means the non-monocot peptide scores highly enough to be included so the group is completely non-specific. Following the same principles, *S* for commelinid-/grass- and grass-specificity were also calculated using highest scoring non-commelinid and non-grass hits respectively.

Different cut-off values for this threshold were investigated using the groups containing the genes from the specific or non-specific test sets. For comparison of the *S* metric with simple pairwise percentage identity, this was calculated from global alignment by MUSCLE of the rice member of the group to its closest non-monocot hit identified by blastp.

### 2.8 Functional annotation of universal groups

To characterize the functions of the set of universal groups and those of the subsets classified by the pipeline as monocot-/commelinid-/grass-specific, functional annotations were obtained. General gene descriptors and Gene Ontology terms from Ensembl Plants were assigned to groups from their member rice and maize peps. Where present, linked publications, gene descriptors, and symbols and trait ontology were assigned to groups from database entries for their member peps taken from RAP-DB (Sakai *et al.* 2013) and KnetMiner-rice for rice and MaizeMine (Shamimuzzaman *et al.* 2020) for maize and KnetMiner-wheat for wheat. Entries were retrieved from web interfaces except for KnetMiner where cereals knowledge graph (Hassani-Pak *et al.* 2021) with programmatic access was used to retrieve gene-TO and gene-GO relations for wheat and rice genes along with supporting publications.

## 3 Results

### 3.1 Identification of highly conserved peptides present in all grasses

Initial steps (Boxes 1–3 in Fig. 2) identified 17 816 groups of similar peps that were present in at least 12 of the 16 grass species from their original gene models present in Ensembl Plants release 56. Of these, 6352 groups passed criteria for universality and high conservation (i.e., had members for all 16 species and minimum *R* > 0.65). However, correct gene models are frequently missing from annotated genomes particularly where there is no transcript information to support these as is often the case for lower expressed genes in less well studied species. Therefore, the genomic sequences were searched for gene models for each group and for each gene model that was missing or low scoring using the genBlastG tool with consensus peptide sequence of the group HMM profile as query (Box 4 in Fig. 2). By incorporating the new gene models identified into groups the number of highly conserved universal groups was more than doubled from 6352 to 13 312 showing the importance of the genBlastG step. The species break-down of the new gene models obtained by genBlastG (Table 2) within these groups shows the newer genomes from *Saccharum spontaneum* and *Lolium perenne* have the most whereas the intensively studied wheat with extensive transcript resources has the fewest.

**Table 2.** Counts of peps or groups for each grass species in universal_grass_peps database.

Grass species	Group members^a^	Members that are genBlastG gene models	Groups with associate peps	Max associate peps in one group	Total associate peps
Brachypodium_distachyon	13 312	347	5605	25	11 787
Hordeum_vulgare	13 312	988	3463	33	7507
Leersia_perrieri	13 312	1353	4983	15	9374
Lolium_perenne	13 312	2218	3514	28	7214
Oryza_rufipogon	13 312	952	4896	16	9475
Oryza_sativa	13 312	1932	3507	22	5729
Secale_cereale	13 312	560	3380	39	9900
Triticum_aestivum	13 312	216	13 114	120	82 480
Echinochloa_crus-galli	13 312	322	12 793	59	46 851
Eragrostis_curvula	13 312	1407	5863	32	11 739
Panicum_hallii_HAL2	13 312	232	4950	26	9496
Saccharum_spontaneum	13 312	2437	8538	36	19 508
Setaria_italica	13 312	843	4886	27	9467
Setaria_viridis	13 312	154	5831	27	12 308
Sorghum_bicolor	13 312	273	5291	36	10 947
Zea_mays	13 312	687	9233	23	26 527

a By definition, all grass species have same number of group members.

The group membership was compared with orthogroups generated by OrthoFinder (Emms and Kelly 2019) from the same set of Ensembl gene models from all 16 grasses. These orthogroups contain peps of divergent function and have unlimited numbers of peps per species but could be used as an alternative starting point for the pipeline. The comparison showed that 91% of universal_grass_peps groups had all their members (excluding genBlastG members) in the same orthogroup (Supplementary Table S4). This finding does suggest that vast majority of group members are all orthologs of each other, despite non-orthologs being allowed. It also suggests that using OrthoFinder rather than Ensembl ortholog predictions in pipeline would have little impact on final group composition; furthermore the Ensembl method considers gene order in a small proportion of predictions. It was therefore decided to keep the Ensembl orthologs as the input to the pipeline (as in Fig. 2).

**Figure 3.**
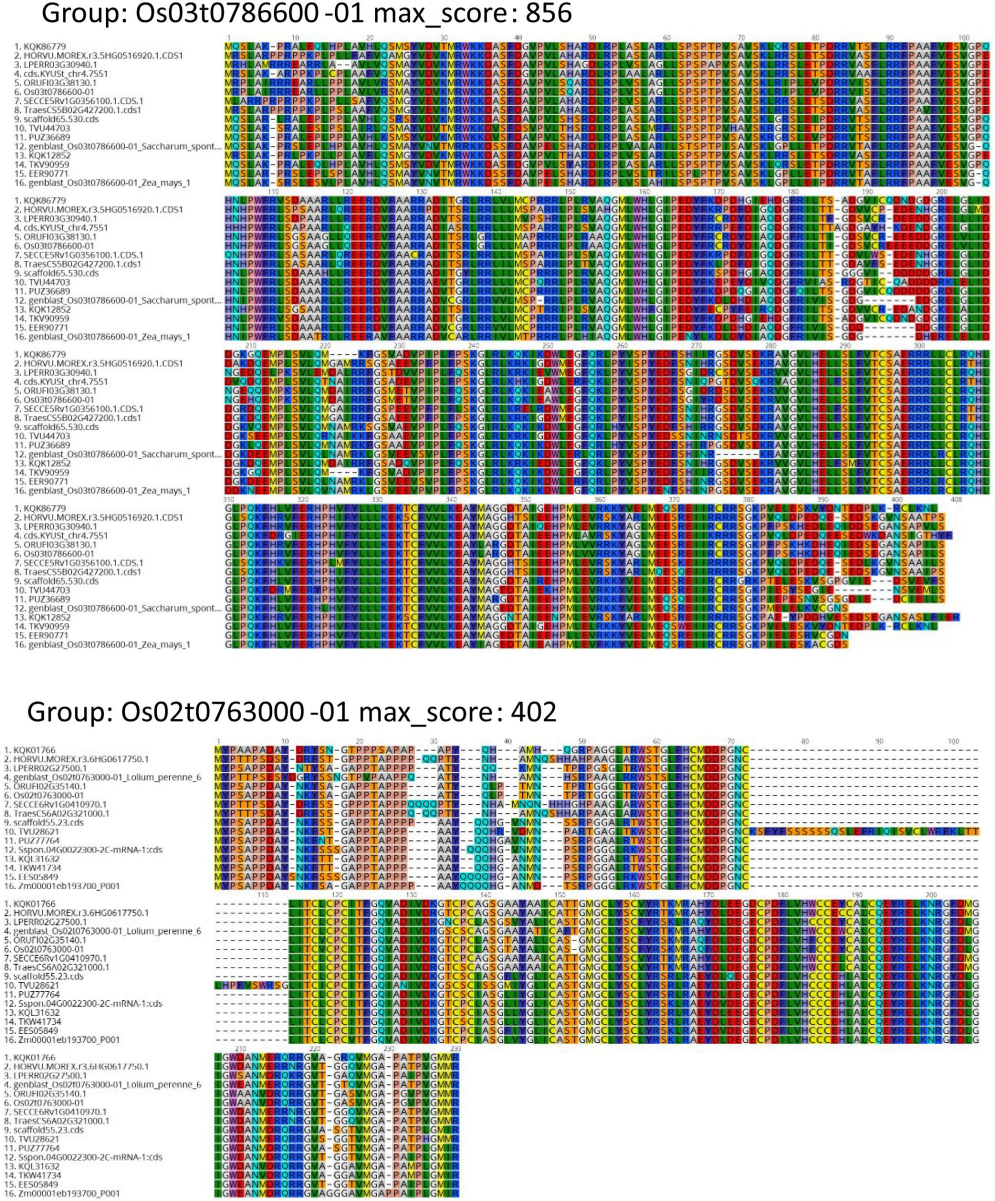
Two example group multiple alignments from the universal_grass_peps set of groups. Sequences are from grass spp 1. *Brachypodium distachyon*, 2. *Hordeum vulgare*, 3. *Leersia perrieri*, 4. *Lolium perenne*, 5. *Oryza rufipogon*, 6. *Oryza sativa*, 7. *Secale cereale*, 8. *Triticum aestivum*, 9. *Echinochloa crus-galli*, 10. *Eragrostis curvula*, 11. *Panicum hallii* HAL2, 12. *Saccharum spontaneum*, 13. *Setaria italica*, 14. *Setaria viridis*, 15. *Sorghum bicolor*, 16. *Zea mays*. Sequences predicted by genBlastG have names starting “genblast” others are Ensembl gene models. Max score is the score of the consensus against the HMM profile generated from the alignment.

The set of 13 312 highly conserved, universal groups form the basis of the universal_grass_peps database. These groups contain sequences that all match the profile well but also contain different degrees of divergence. Two example multiple alignments used to generate the HMMs for two groups are shown in Fig. 3. These show high conservation including for the novel genBlastG gene models. The second group, Os02t0763000-01, illustrates a complication found in many groups—a section of sequence found only in one species.

### 3.2 Validation steps with genomes from outside Ensembl Plants

The novel genBlastG gene models found by the pipeline were not originally annotated in the genomes so could be pseudogenes. This was tested using the new gold standard IC4R annotation of the rice genome which has improved gene models developed using multiple RNAseq datasets (Sang *et al.* 2020). Out of 1932 genBlastG rice gene models, 1477 (76%) have matches in IC4R gene models with >90% coverage of query gene model on correct chromosome; these matches average 93% peptide sequence identity (Supplementary Table S5). This suggests that the great majority of the genBlastG predictions are not pseudogenes and will be confirmed by future evidence.

**Figure 4.**
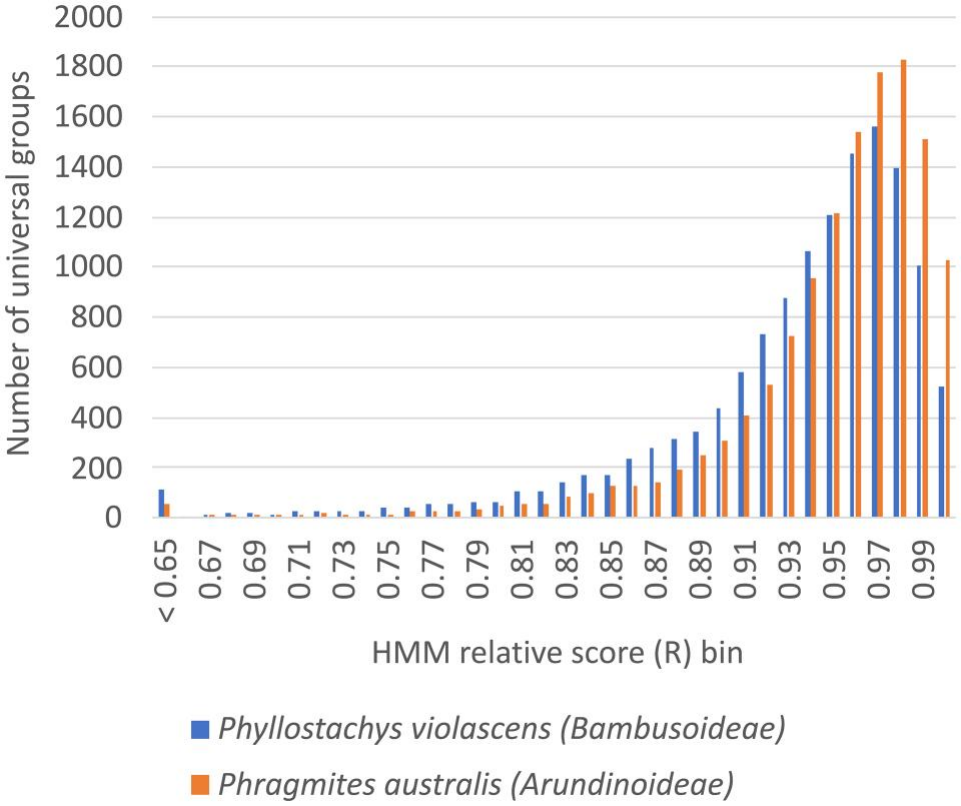
Distributions of *R* scores of top hits found in newly sequenced grass genomes from *Bambusoideae* and *Arundinoideae* clades to each of 13 312 universal_grass_peps group HMMs.

As universal_grass_peps database is derived from Ensembl Plants grass genomes, it could contain groups that do not have corresponding genes in the major grass clades absent from Ensembl Plants (Fig. 1). To test this, genomes recently available on NCBI from two of these clades, *Bambusoideae* and *Arundinoideae*, were used to look for matches to the universal_grass_peps HMMs and the distributions of the resulting *R* scores are shown in Fig. 4. Of the 13 312 universal groups 99.2% and 99.6% had matches (*R* > 0.65) in the *Bambusoideae* and *Arundinoideae* species, respectively (Fig. 4; Supplementary Table S6). All but 1.2% of the universal groups had matches in both species and are therefore supported as universal (The 1.2% which were not supported by these genomes are clearly flagged in the universal_grass_peps database and can be easily filtered out if desired).

### 3.3 Matches of grass genes to universal groups

All grass gene model peps from the 16 Ensembl genomes were searched against the HMM profiles of universal_grass_peps for hits with *R* above the cut-off of 0.65; if these are not the member of any group they are classified as associated to the group (Box 5 in Fig. 2). The total number of associated peps for each species is shown in Table 2 and generally reflects the degree of gene duplication. The grass pep hits are also used to define a total of 920 supergroups of closely related function (Supplementary Table S7). Supergroups can contain groups with same molecular function but differing regulation due to sub-functionalization.

### 3.4 Monocot-/commelinid-/grass-specificity

All peps from the 58 non-grass species in Ensembl Plants were scored against the HMM profiles of universal_grass_peps (Estimate Specificity block in Fig. 2). The results were used to derive the specificity metric *S* for each group, where *S* is minimum *R* value from group members minus maximum *R* value for any peptide from non-grass to give monocot-/commelinid-/grass-specificity. Distinguishing between monocot-specificity, commelinid-specificity, and grass-specificity is dependent on maximum *R* values from only three species (two non-grass commelinids and one non-commelinid monocot) so these sub-classifications are less secure, and the overall monocot-/commelinid-/grass-specificity is emphasized here.

**Figure 5.**
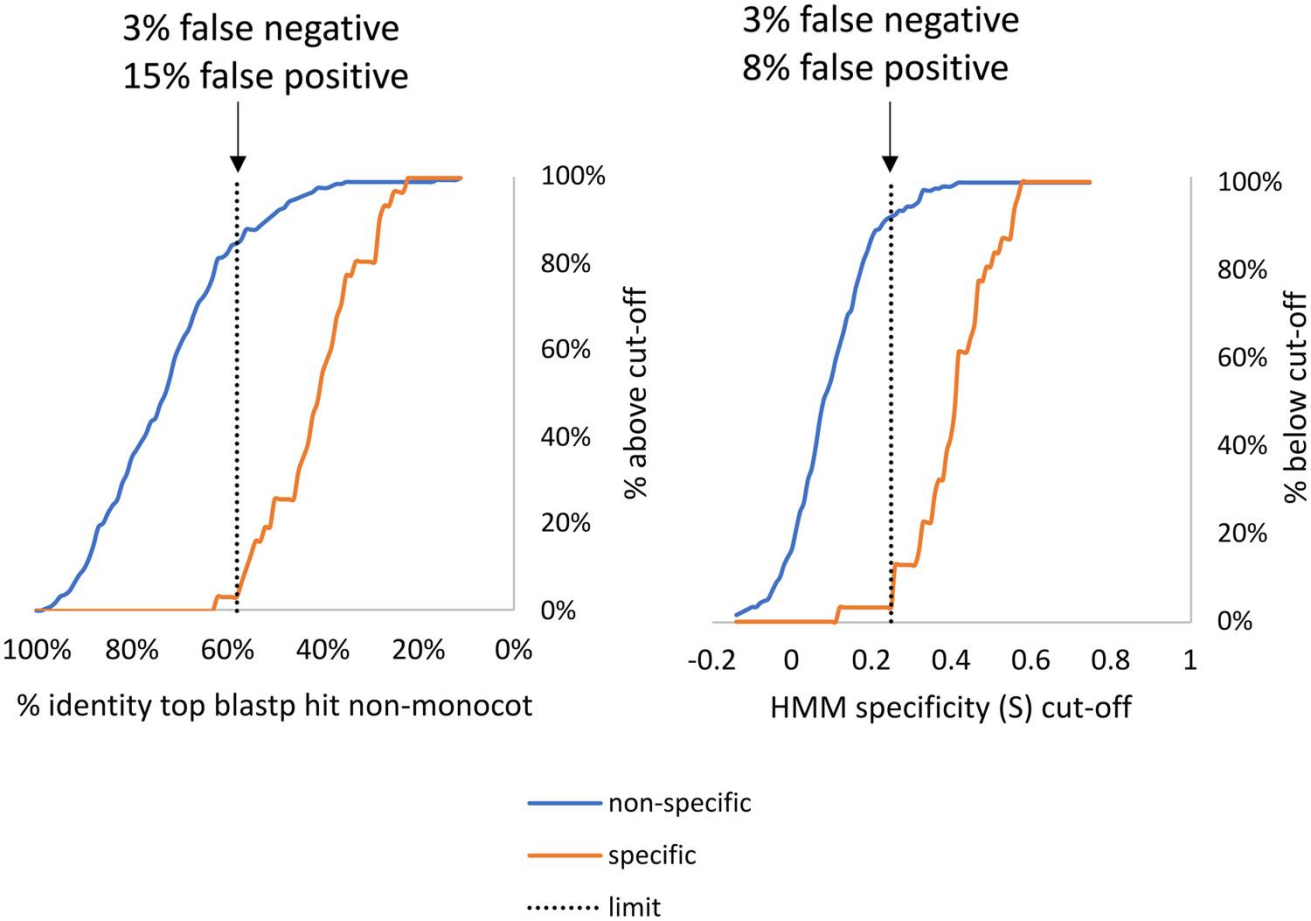
Proportion of groups of genes expected to be of non-specific function or specific function for commelinid/grass species that pass varying cut-off thresholds for two metrics of specificity. Left panel: percentage identity of closest non-monocot hit to rice member of group. Right panel: *S* metric defined as lowest HMM relative score of group member minus the top relative score for a non-monocot hit. In both panels, the selected limit is shown with the corresponding false negative and false positive rates.

The *S* metric is a measure of sequence divergence from the grass profile that can be used as a basis for an initial hypothesis of function divergence in the same way that other sequence-based measures are used. The test sets were used to gauge the performance of *S* as a means of determining specificity, i.e. the non-specific test set of 215 peps expected to have common function in all plants (Supplementary Table S2) and the specific test set of 31 peps with commelinid-/grass-specific functions (Supplementary Table S3). The *S* metric was compared with simple pairwise percentage identity with the best non-monocot hit for these sets (Fig. 5). *S* performs better than pairwise identity at discriminating between the two sets as choosing cut-offs with same false negative rate of 3% (<58% for pairwise identity, and >0.25 for *S*) gives more false positives at 15% for pairwise identity than for *S* at 8%. As only 34 known specific genes were used, the 3% false negative represents a single gene, Lsi6. The low *S* score for Lsi6 is partly due to a low minimum *R* value for the group due to a *S. spontaneum* pep which is shorter than all others in the profile and could be due to an erroneous gene model (Supplementary Fig. S1).

Applying the cut-off S of >0.25 which gave 8% false positive and 3% false negatives with the test sets (Fig. 5) to the complete set of 13 312 groups gave 4609 defined as monocot-/commelinid-/grass-specific. This set was divided into subsets classified as probably monocot-specific (394 profiles), commelinid-specific (1411 profiles), and grass-specific (2804 profiles) based on values of *S* calculated from best hits for each taxonomic level and is listed in Supplementary Table S8.

### 3.5 Functional annotation of universal groups

Functional annotations for all universal groups were derived from public annotations of their rice, wheat, and maize members in RAP-DB, KnetMiner-rice, KnetMiner-wheat, and MaizeMine. The retrieved descriptors, GO terms, associated publications, and TO terms for each group are available in the universal_grass_peps database. The subset of 4609 groups classified as monocot-/commelinid-/grass-specific by *S* > 0.25 is also available in Supplementary Table S8. Most of these groups have no linked publications and only general descriptors and high-level GO terms based on domains. Of all the biological process GO annotations, most are assigned to at least one group suggesting there are some monocot-/commelinid-/grass-specific aspects of most processes in grasses. The processes that are dominated by these specific genes are shown by the terms which are enriched; there is clear enrichment of groups of regulatory proteins, especially those involved in control of transcription and of protein activity (Supplementary Table S9). Enriched molecular function GO terms are mostly DNA-binding and enzyme activities; the most enriched enzyme category is hydroxycinnamoyl transferase activity which may reflect the importance of these moities on lignin and xylan polymers in grass cell walls (Chandrakanth *et al.* 2023). The traits defined by TO terms in Supplementary Table S8 are associated with variants of the member rice, maize, and/or wheat genes from evidence in the associated publications. Particularly common traits affected are grain size (70 groups), flowering time (50 groups), with numerous morphology traits as might be expected. However also common are traits for insect/pathogen defence and abiotic stress resistance.

## 4 Discussion

Genes of common function that occur in all species within a taxonomic unit (universality) indicate that the function is likely key to fitness. Although sequence similarity is a measure of likelihood of shared function, using existing bioinformatic resources it is not straightforward to compare genes in a systematic way, nor to check for criterion of universality given variation in completeness of genome annotation. The new approach described here provides predictions of all universal grass genes with putative common function and estimates of their specificity to monocots/commelinids/grasses. The requirement for universality led to the incorporation of the genBlastG step to find missing genes (Box 4 in Fig. 2); this step generated 14 921 new gene models. The fact that 76% of the rice genBlastG gene models were found in the high-quality IC4R rice genome (Supplementary Table S5) suggests most of these new gene models are not pseudogenes and that future grass RNAseq studies will validate them. The use of a metric based on HMM profile score to estimate how specific the function of a universal group is to monocot/commelinid/grass species is another novel aspect of the pipeline; it provides a systematic basis for an assertion of such specificity for genes of unknown function.

### 4.1 Importance of monocot-/commelinid-/grass-specific genes

Grasses typically have a haploid set of about 40 000 protein-coding genes. The analysis here indicates that about 13 000 of these are universal in grasses and that about a third of universal genes are monocot-/commelinid-/grass-specific. All of the known function specific genes (Table 1) are in the specific set of 4609 genes in universal_grass_peps (except Lsi6) so the set likely includes other genes involved in these known monocot-/commelinid-/grass-specific traits (stomata, cell walls, Si deposition, etc) and any linked literature is listed in Supplementary Table S8. An analysis of GO terms (Supplementary Table S9) suggests many more biological processes, in fact almost all GO biological process terms, involve some genes which are monocot-/commelinid-/grass-specific. The specific genes are particularly enriched for regulatory functions as might be expected given the radically different organization and morphology of grasses.

The importance of variants of the monocot-/commelinid-/grass-specific genes for crop traits is seen from publications associated to the identified sets (Supplementary Table S8) including numerous variants associated with grain yield, abiotic stress and defence. Where a trait is known to be commelinid- or grass-specific, the classifications generated here can help to identify candidate genes involved in the trait. In our own work on dietary fibre QTLs in wheat grain, candidate genes identified as likely commelinid-/grass-specific were prioritized as dietary fibre is mostly feruloylated arabinoxylan (AX) that only occurs in commelinid species. The causal allele was eventually shown to be a variant of one such gene—a commelind-specific peroxidase involved in cross-linking AX (Mitchell *et al.* 2023).

### 4.2 Limitations of approach

All high-throughput predictions of shared function based almost entirely on peptide sequence need to be used with caution and cannot substitute for detailed knowledge of the particular protein. A major problem for any pipeline trying to find universal genes is the large variation in sequence conservation of proteins of same function, e.g. different subunits of cytochrome c oxidase showed conservation varying from 16% to 64% in comparison of yeast and animal sequences (Das *et al.* 2004). Use of HMMs rather than whole-sequence similarity helps as non-conserved regions contribute less to score but even the HMM derived *R* score showed considerable variation in the curated non-specific gene set. The cut-off of 0.65 which 90% of this set passed was chosen to be inclusive so that nearly all true universal genes will be found but this low threshold does mean that there will also likely be “universal” groups containing genes with similar but non-identical functions.

The approach here should therefore be treated as a first best guess of shared function similar to comparing percentage identity (as biologists often do as a first step) but more likely to be accurate (as evidenced by the better discrimination between the specific and non-specific function sets; Fig. 5) as the HMM approach weights the conserved parts of sequence important for function, exploiting the fact that the identified genes are highly conserved and present in all grasses. As stated above, the universal gene groups would be expected to include nearly all cases of genes which have identical function in all grasses, but they can also include cases where there are highly similar functions with divergent aspects. Therefore, the next step after identifying a group of interest should be to inspect the multiple alignment (as in Fig. 3 and Supplementary Fig. S1) to judge the extent of divergence in different grasses; all 13 312 multiple sequence alignment files are available in the universal_grass_peps database.

Inspection of these multiple alignments can also highlight outlier sequences (as in Supplementary Fig. S1) which may be due to incorrect gene models. The criterion of universality does make this pipeline highly dependent on the quality of the grass genome assemblies and their annotation. Better genome quality and annotation will help to check and improve universal_grass_peps as shown by use of rice IC4R genome (Supplementary Table S5).

### 4.3 Uses of universal_grass_peps database

Where experiments reveal large sets of grass genes or peps such as transcriptomics, proteomics, or genes underlying QTLs, they are inevitably dominated by genes with little or no information on function. Even for rice, the most studied grass, only 13% of genes in RAP-DB database (Sakai *et al.* 2013) have associated publications and only a minority of these publications specify function. For such unknown genes it is useful to have a systematic approach to identifying those that are of grass-/commelinid-/monocot-specific function as this information can point to the nature of the process they are likely involved in. For example a network of co-regulated genes identified from transcriptomics enriched for grass-specific functions indicates involvement of the network in a grass-specific trait such as inflorescence development, Si deposition etc. Using the look-up tables available in universal_grass_peps, any set of grass genes from the grass genomes used here can be used to find all those in, or associated to, the universal groups. For each group identified, the values of *S* metrics as measures of specificity are given along with resulting categorization as likely non-specific, monocot-, commelinid-, or grass-specific providing some evidence on role of gene.

### 4.4 Future developments

The pipeline reported here is a first attempt to implement the concept of using universal genes to identify groups of putative common function and likely specificity but could be improved upon in future. Improvements might be made by using recently released alternative packages for finding gene models in genomes such as miniprot (Li 2023) (to replace genBlastG for Box 4 in Fig. 2) with reportedly better performance. The validation steps here show that future updates with more diverse and better-quality grass genomes will improve universal_grass_peps. Further in the future, whilst HMMs are a convenient and fast way of obtaining profiles for groups, they are actually a proxy for comparison of structures; potentially a better approach would be direct comparison of predicted structures such as that generated by AlphaFold although such a capability is not currently available.

## Supplementary Material

vbaf079_Supplementary_Data

## Data Availability

The universal_grass_peps database is available at https://data.rothamsted.ac.uk/dataset/universal_grass_peps.
